# American and Southern Dental Associations

**Published:** 1888-11

**Authors:** 


### PROCEEDINGS.

### American and Southern Dental Associations.

### JOINT SESSION.

[Reported for the DENTAL REGISTER by "MRS. M. W. J."

The American and Southern Dental Associations met in joint session, in Louisville, Ky., Aug. 28 to Sept. 1, inclusive.

Separate business meetings were held by the two associations, the joint meetings being devoted exclusively to scientific discussions. The business transacted was the usual routine. In both associations a resolution was adopted to memorialize Congress to remove the tax on dental goods of every kind.

A resolution was adopted in the American Association declaring it non-professional for a dentist to place on his card any thing more than his name, title, and address.

In the Southern Association steps were taken to raise a fund for defence in the suits of the International Tooth Crown Co., as suits are being brought in all parts of the country for infringements of their patents in the past, and licenses and royalties in the future it is desirable to make a few test cases.

Memorial resolutions were adopted in honor of Dr. Geo. W.

Keely, Oxford, Ohio, late Treasurer of the American Association; also Drs. Stoddard Driggs, J. H. DeVore, Wm. Dutch, and C. P. Fitch. Dr. J. H. Prewitt, late 1st Vice President and Dr. H. M. Grant, Abingdon, Va., a former Vice-President of the Southern Association; also Drs. J. S. Franklin and J. H. Cook.

ELECTIONS.

Saratoga was chosen as the place of meeting of the American Association, and the following officers were elected:

President, Chas. R. Butler, Cleveland, Ohio; First Vice-President, A. W. Harlan, Chicago, Ill.; Second Vice-President, S. A. White, Savannah, Ga.; Corresponding Secretary, F. A. Levy, Orange, N. Y.; Recording Secretary, Geo. H. Cushing, Chicago, Ill.; Treasurer, A. H. Fuller, St. Louis, Mo.

Executive Committee: E. T. Darby, Philadelphia, Penn.; G. W. McElhaney, Columbus, Ga.; J. N. Crouse, Chicago, Ill.; Frank Abbott, N. Y.

Galveston, Texas, was chosen as the place of meeting for the Southern Association. The following officers were elected:

President, J. Y. Crawford, Nashville, Tenn.; First Vice-President, Jno. C. Storey, Dallas, Tex.; Second Vice-President, Wm. N. Morrison, St. Louis, Mo.; Third Vice-President, J. S. Thompson, Atlanta, Ga.; Corresponding Secretary, D. R. Stubblefield, Nashville, Tenn.; Recording Secretary, M. C. Marshall, Little Rock, Ark.; Treasurer, H. A. Lowrance, Athens, Ga.

Executive Committee one year: Dr. A. A. Dyer, Galveston, Tex.; Dr. W. R. Clifton, Waco, Texas.

Two years, Dr. G. S. Staples, Sherman, Tex.; Dr. B. H. Catching, Atlanta, Ga.

Three years, Dr. H. E. Beach, Clarksville, Tenn.; Dr. H. J. McKellops, St. Louis, Mo.

JOINT MEETINGS.

Two joint meetings were held daily, presided over alternately by Dr. Frank Abbott, President of the American Association, and Dr. B. H. Catching, President of the Southern Association.

The first joint meeting was called to order at 11 a. m. Tuesday August 28, and opened with prayer by the Rev. Mr. Moore.

A brief address of welcome from Mayor Jacobs, was responded to by Dr. A. O. Rawls, on behalf of the Kentucky State Dental Association, Dr. E. T. Darby, on the part of the American Association, and Dr. J. Y. Crawford, for the Southern Association.

The meeting then adjourned to 7:30 p. m. when the Presidents of the two Associations delivered their addresses. Both addresses dealt with the subject of Dental Education, though under different aspects of the question. Dr. Abbott reviewed the advances made by dental surgeons in the past few years, through the increased facilities for thorough dental education, and the knowledge gained through microscopical researches.

Dr. Catching, on the other hand, claimed that, through lack of special medical training the average dentist is not entitled to the position accorded him of " Specialist in medicine."

He takes the position that the time has come to do away with separate dental schools, and special dental degree; that a medical course should precede admittance to the dental infirmary. He considers that under the present system of education the number of dentists who practice their profession as specialists of medicine is very small compared to those who follow it as a trade. They are not competent to perform the surgical operations properly lying within their province, nor to follow out the subsequent line of treatment. In case of prosecution for malpractice they have no defence.

This paper called forth a very animated discussion in which the many college professors present took prominent part, in defence of the present system of education.

In regard to the legal aspect of the case it was generally agreed that it was not a question of schools or of degrees but of skill and competency. In closing the discussion Dr. Catching said that in case of fatal result from the administration of chloroform at the hands of a physician he has only to say " heart failure" and he is all right, but if it is the patient of a dentist, he has to call in a physician to give a certificate, and the iron bars are very apt to receive him.

Before adjourning, invitations were announced from the Polytechnic Library Association and from the Commercial Club, offering the freedom of their rooms to the members of the associations, and calling attention to various points of interest in and around the city of Louisville. Also an invitation from the latter club for a steamboat excursion on the Ohio River.

The joint session was called to order again at 9:30 a. m. Wednesday. B. H. Catching, in the chair, called for the report of the Committee on

OPERATIVE DENTISTRY.

Geo. H. Winkler, Southern Association; E. T. Darbey, American Association, Chairmen.

In the absence of Dr. Winkler, to whom the report had been allotted, Dr. Darby reported four papers accepted. The subjects which have attracted most attention during the past year, are Immediate Root-Filling, Implantation, Copper Amalgam, and the Treatment of Pulpless Teeth. The scientific use of germicides has so lessened the dangers of septic infection that great risks are now safely taken. Implantation, so far, is an apparent success, for the time being, but time alone can settle the question of its permanent results. Copper amalgam has been in use for some time in Europe, but it is only within the last two years that it has been much used by American dentists. Those who use it most report very satisfactory results.

The first paper was read by Dr. W. Storer How, describing the method of using

PORCELAIN INLAYS,

Or stoppers for cavities of decay, more especially in the labial surface of incisors. The paper was illustrated by a number of very perfectly executed samples which were passed around for inspection. The details of manipulation are very clearly described in the *Cosmos*, August, 1887.

*Dr. J. J. R. Patrick*—Next read a paper on the Method of Setting Artificial Crowns. He said that all roots of natural teeth are available for the adjustment of artificial crowns, by means of a gold band between the gum and the root, but every thing depends on a close fit of the band to the root. The difficulty lies in the fact that we are dealing with the base of a cone a band when driven down standing off more or less at the lower edge. To overcome this difficulty the root must be beveled a little on the labial side over which the band can be slipped, the other side being undercut.

A porcelain crown with platinum band baked in it, which can be telescoped inside of the gold band of the root, offers many advantages. The choice of metal for these operations is of great importance; it must have both pliability and stability.

Dr. Patrick gave the following formulæ:

| | | |
|---|---|---|
| CROWN GOLD. | Pure gold | 15 |
| | Platinum | 1 |
| | Cyanide of potassium | 5 |

Kept at the fusing point thirty minutes.

| | | |
|---|---|---|
| BAND GOLD. | Pure gold | 1 |
| | Coin gold | 1 |

Fuse.

| | | |
|---|---|---|
| SOLDER No. 1. | Band gold | 89 |
| | Copper | 4 |
| | Soda-borax | 10 |
| SOLDER No. 2. | Solder No. 1 | 89 |
| | Silver | 7 |
| | Copper | 4 |
| | Soda-borax | 7 |

All gold solder must be made of the gold it is to be used with, that it may have the same color and texture. To crown a root which is fractured below the gum, a platinum band is placed around it, rising above the margin of the gum to hold away the soft tissues and filled with cement, the gold crown or band being telescoped over the platinum band. For implantation he would prefer an artificial tooth, which in a healthy mouth would be retained by a process of proliferation.

*Dr. C. E. Kells, jr.*—Read a paper on the Conductivity of Filling Materials, illustrated by a series of experiments with a very delicate thermostat, and a series of tiny cells made respectively of the enamel shell of a molar tooth, and cells similar in size and shape made of gold, tin, copper, amalgam, ordinary alloy, agate cement, oxy-chloride of zinc, and gutta percha filling materials. These cells were successively placed on a small zinc disk, almost in contact with an electric bell connection, and filled with warm water, the expansion of the material completing the circuit and ringing the bell. With the enamel cell there was no ring; with the gutta-percha a ring only after a long wait; with the gold cell the ring was instantaneous and also with the amalgams, and only a short wait for the cements, the various filling materials thus falling into three groups—1st, the metals, 2nd, the cements and 3rd, gutta-percha, even the latter not being an absolute non-conductor.

*Dr. H. Gilmer*, Quincy, Ill., read a paper with blackboard demonstration, of the method of constructing

CHEAP CROWNS.

We all desire to do the greatest good to the greatest number of patients, and we all have to face the fact that a great many of our patients are not able financially to have the expensive gold and porcelain which are our pride. For them we must have a crown which can be produced at low cost, both for materials and time: For this purpose we can use a platinum band and Weston's metal for the posterior teeth, while for the anterior teeth a crown with gold band and porcelain face is quickly and cheaply produced by the following method: A platinum band is fitted to the prepared root and filled with modelling compound, and the teeth closed to give the articulation. Removing carefully this is inserted in plaster. When set and dried, the plaster is drilled, the wax removed and Weston's or Watt's metal poured, using chloride of zinc as flux, to attach the metal to the band. When necessary a dowel is fitted to the root canal and removed with the modelling compound. In this way a serviceable crown is easily, quickly, and cheaply made for those who can not afford any other.

The subject of Operative Dentistry, in connection with the four papers read was declared open to discussion. The methods of Drs. How, Patrick, and Gilmer were passed without discussion. The experiments of Dr. Kells were pronounced ingenious and interesting, but proving only what is universally admitted.

*Dr. C. N. Pierce*—Said that though it is true, that the materials vary in conductivity as shown in the experiments, the physiological or pathological condition of the tooth is entirely ignored. Gutta-percha though the best non-conductor is only a mechanical covering for an exposed pulp; it has no action on the tissue, but retains all exudations and thus increases inflammatory conditions. If we first cauterize the pulp we get a non-conducting surface which can be covered and saved. If there were absolutely no exudations we might save with gutta-percha, but we can save from fifty to seventy-five per cent. with other materials

*Dr. Kells*—Said he had made no reference to capping exposed pulps with gutta-percha or any thing else. If he were to cap one hundred pulps he would expect to have one hundred failures. Even if he only saved seventy-five, he should feel certain that the other twenty-five had gone to some one else. His experience had taught him that exposed pulps should be destroyed. If the pulp has nature's cap, a layer of dentine, even though very thin, he would cover it carefully with thin oxy-chloride of zinc and carbolic acid; then a harder oxy-chloride cement, then gutta-percha and then a metal surface filling, having it four stories high! He doubted whether the cauterized pulp surface was a non-conductor. Some persons can't raise an abscess but can tolerate a dead tooth ten or fifteen years.

*Dr. Patrick*—Does not think it possible for a ruptured pulp to recover—there are no absorbents in the pulp. It has never been proved that they are saved; it is contrary to experience; contrary to physiology.

Drs. Atkinson, Darby, Allport, Dr. W. H. Morgan, Freeman, of Nashville, and others cited remarkable cases where they had positive evidence that the pulp had been saved alive for years, by capping—in one case the same pulp having been capped over three exposures, from different cavities of decay, at different dates.

Drs. Patrick, Cravens and others considered that the function of the pulp was fulfilled when the tooth is fully formed, its work after that being towards self-destruction, choking itself out by deposits of secondary dentine in the roots and pulp chamber.

Dr. Taft said that the destruction of the pulp would result, sooner or later, in the destruction of the tooth. The tooth would break down, through loss of continuity of structure, independent of decay. Teeth with devitalized pulps are also more subject to ordinary decay than teeth with living pulps. The tooth also deteriorates under attrition, comminutes and wears away. The dentine is a living tissue, and requires continued supplies of nutrient material; it retains its sensitiveness only through the pulp. Its function does not cease with the completion of the tooth—and who can say when it is complete? Changes are continually going on; it is becoming more dense, the tubuli filling up through the function of the pulp. If it was a worthless thing nature would cast it off. As to the preservation of an exposed pulp, no surgeon undertakes to treat all cases alike, there must be the ability to discriminate conditions. A good knowledge of systemic conditions must be possessed. We must learn how long the pulp has been exposed, whether it is the result of accident or of decay; whether it has beeu subject to irritation or inflammation. If the conditions are all good, exposed pulps can be saved, but much depends on the manipulative ability of the operator.

He was sorry to hear so many say they had abandoned this conservative practice. He perhaps discriminates more closely than formerly, but wherever it appears at all feasible he tries to save the pulp alive.

Dr. E. T. Darby said he had intended to enter his protest, but Dr. Taft had taken the words out of his mouth, and said it better than he would have done. At the request of the President, Professor Darby gave his method of capping, which he has not varied for twenty-five years, using oxide of zinc with creosote next the pulp, then oxychloride, and filling usually with gold. He described a tooth filled for a gentleman in the Navy. A bad exposure was capped in this way, and the cavity filled with gold. Six years later he returned from a voyage with a decay on the distal surface with exposure of living pulp; it was again capped and filled with gold. Three years later there was decay of the buccal surface, with the pulp exposed and bleeding. He capped it again, and to-day that pulp is living, with three capped exposures. He considers gutta purcha the worst material that can be used.

Dr. Patterson said that one important factor had not been touched upon, and that was *the patient*. We must consider not only our own triumphs over difficulties, but the way in which we can give the greatest comfort and the greatest benefit to the patient. We don't think enough of the objective end of the forceps.

Dr. Freeman said that if the vitality of the individual was poor, a pulp in pathological condition would be lost. There are certain conditions in which the pulp cannot be saved, and again there are other pulps that cannot be destroyed with arsenic; they resist all irritation. Dr. Freeman does not agree with Dr. Taft as to the deterioration of pulpless teeth. The dentine is covered with the cementum, which is nourished and kept in health by the pericementum.

This closed the discussion of Operative Dentistry, the hour for adjournment having arrived.

7:30 P. M., Wednesday, President Abbott in the Chair.

HISTOLOGY AND MICROSCOPY.

Dr. Frank Abbott, American Association; Dr. Jno. G. McCulloch, Southern Association, Chairmen.

Dr. Patterson occupied the Chair while Dr. Abbott made the re-report. He said that there was but little to report during the past year except the continuation of the work of Drs. Heitzman and Bodecker on the development of the teeth, the investigations of Dr. Andrews, and the investigations of Dr. G. V. Black in Bacteriology. The work of Dr. Black summarizes the results of eight years of patient investigation, and cannot be too highly commended.

The Committee presented two papers, one from Dr. I. P. Wilson, Burlington, Iowa, entitled:

THE APICAL PORTION OF THE CEMENTUM PHYSIOLOGICALLY AND PATHOLOGICALLY CONSIDERED.

The paper was quite short. The structure of this portion of the cementum, as revealed by the miscroscope, was described, with the effects of increased or diminished supplies of blood upon the functions of the surrounding membrane, in its relations to the apical portion of the tooth. The main point of interest is that, although the devitalization of the pulp in itself does not interfere with the vitality of the cementum, yet the methods employed may have this effect, with resulting necrosis. After the root canals have been filled for a time, the tooth may become tender on pressure, with a sensation of uneasiness; this is from one of two causes; either the canals are imperfectly filled, or arsenic has been injudiciously used, occasioning the death of the cementum. Nature disposes of this through solution of the lime-salts. For this reason arsenic should be used with great caution. If the first application fails, there is danger in the second or third application. Many incurable abscesses are the result of arsenical poison, and the paper was intended to call attention to these facts in the hope of leading to safer practice.

*Dr. Atkinson* characterized this paper as *an awakener*, bearing closely on the occult movements in nutrition and denutrition. From the results described in the paper, men are justly getting afraid of arsenic, and yet they can't see their way to give it up. It is strange that men are not more alive and awake to such investigations, but the revelation of truth only comes through our necessities, then the light flashes in. Men don't distinguish between inflammation and activity--oxygenation. Who knows how arsenic acts? We take hold of things fearfully, because we don't know, but are ashamed to say we don't know. Arsenic acts through the affinity of arsenious acid and the protoplasmic tissue in the pulp, the semi-fluid portions of the pulp. If only the legitimate quantum is used, it will sleep till Gabriel's trump. We should not wilfully kill babies to have funerals—pulp babies—there is bungling somewhere. There is a very strong conviction that microbes enter into tissue territory and set up a retrograde ferment—that microbes produce pus--they make a hell, raise Ned! Learned talk, but it says nothing. What is practical? What is the evidence? The prophet of old prayed that he might be delivered from the memory of his former sins; but we have had a better illumination; we want to remember, that we may do better, and avoid former errors.

*Dr. Storey*, Dallas Texas, said he had, for more than twenty years, been trying to learn the better way, that he might do without arsenic, and he hoped some one would tell him now. After some further remarks by Dr. Atkinson, on the method of relieving soreness and tenderness of a tooth, by pressure, producing arrest of the neural current, the discussion was closed.

Dr. C. R. Butler, Vice-President of the American Association took the chair, while Dr. Abbott read a paper entitled:

THE ODONTOBLASTS IN THEIR RELATION TO DEVELOPING DENTINE.

Dr. Abbott reviewed the *Secretion theory* and pointed out wherein it failed to account for the facts observed. He then defined the *Transformation theory*, showing how much nearer it comes to solving the mooted questions.

The discussion of this paper was confined to Drs. Sudduth and Abbott, the former upholding the Secretion theory.

*Dr. Sudduth* said that Dr. Abbott made no distinction between the cortical and cancellous portions of bone, which the secretion theory alone explains. In cancellous bone each cell is a separate individual; in the cortical portion, the cortical sheath is laid down by a membrane which secretes it in layers; it is secreted with regularity, and only in certain lines, certain places. Dr. Sudduth also considered that Dr. Abbott ignored the nuclear structure of tissues. He had visited the laboratory of Drs. Bodecker and Heitzman, and spent half a day examining their specimens, but could not see how they were able to draw their conclusions from the specimens he saw, which were all miserably stained. In carfully stained specimens the nucleus can be clearly seen, showing the return of tissues to their embryonal condition. The study of the secretion of shell substantiates the theory of the secretion of enamel, dentine and other tissues.

The slides sent to the Boston meeting by Dr. Miller, from Berlin, show these points very clearly.

*Dr. Abbott* said if we did not differ in opinion we should never learn anything. We look at the same thing with the same glass, but see and understand in different ways; no two see alike. The mind is pre-occupied with certain ideas, we have our pre-conceived opinions, and in looking for what we expect to see, we fail to see what appears plain to others. With a glass of high power there is discovered in every odontoblast a reticulum full of little nuclei, but this is only a provisional step in the formation of dentine. They do things wonderfully well in Boston, and showed wonderful specimens, but all are not bound to agree and see the same way, even if the specimens are from Berlin. Dr. Abbott spoke at some length on the appearance of sections of carious dentine and its formation.

*Dr. Sudduth* said that no theory ever yet advanced explained the production of the cavity except the germ theory of Dr. Miller—the digestive ferment breaking down tooth substance. Nitric acid dissolves the lime-salts, but the form of the tooth is left intact, until the digestive ferment of micro-organisms—the chromogenic bacteria of the human mouth throwing out lactic acid forms the cavity. Semi-decalcified dentine is the media in which they live. A tooth that is isolated will not decay; there must be a lodging for the microbes. Dr. Miller stands king in this line of investigation, and as time rolls on he will get the credit due him.

*Dr. Abbott* said that teeth do not decay by the disolving out of lime-salts; under the excavator we find it in the semi-decalcified dentine, but broken down; leathery dentine is full of micro-organisms; in the cavity everything is gone. Do the microbes show it up? There are always organisms in the mouth, as everywhere else; they do not destroy us because conditions are not favorable. In diseased conditions of the throat, lungs, liver, kidney, they are always there; this is no less true of the teeth, but if they are kept perfectly clean they will not decay,

*Dr. Sudduth.*—But how is the cavity formed?

*Dr. Abbott.*—In carious dentine, there is as much lime-salts as ever, but it is dissolved. The cavity is formed by acids, whether from decomposing foods or from microbes is a matter of indifference. The living tissues become irritated; the limesalts are displaced, melted down, through the inflammatory process; embryonal tissue is formed. Acids dissolve the lime-salts, and they are taken away.

*Dr. Sudduth.*—How are they taken out? The form of the tooth remains the same.

*Dr. Abbott.*—They are taken away the same as any other waste product, broken down and washed out.

*Dr. Sudduth.*—The admission that the breaking down is due to microbes is the very thing claimed. A tooth may be placed in any putrifying mixture, but it will never produce a cavity, and there is only one form of microbe that will do it, and it is present wherever there is the proper food for it to live on.

*Dr. Abbott* denied that he had made any such admission.

*Dr. Allport* stated that Dr. Boedecker had placed arsenic in a cavity of a tooth which was entirely protected from microbes, yet the tubuli became enlarged and the tooth structure wasted away to the pulps. The swelling of the contents of the tubuli broke down their walls, and decomposition of the contents produced acids, but there were no microbes present. It was the result of inflammatory action.

No reply was made to this, and the discussion was closed.

### MATERIA MEDICA AND THERAPEUTICS.

Jno. C. Storey, Southern Association; A. W. Harlan, American Association, Chairmen.

*Dr. Story* offered the report from this committee. He gave a history of a case in which very peculiar effects followed the hypodermic injection of cocaine. The patient became alarmed at a peculiar sensation of stiffening in the joints of fingers, elbows and shoulders, and left the chair, and soon became unconscious, remaining so from 6 to 11 p. m. He then passed what he called "an awful night" from peculiar sensations, though free from pain. The next day, from his elbows to his wrists broke out a solid mass of pimples, with pus formation, lasting three days and nights, and followed by boils and sores for five days more. His general health was not affected except from weakness.

*Dr. A. W. Harlan* read a paper from *Dr. Arthur C. Hugenschmidt* on

### HYPODERMIC INJECTIONS OF MURIATE OF COCAINE.

This drug has proved very satisfactory in his hands, in over 200 cases, using it several times a day, for extractions, exploring fistulous tracts, exploring for missing teeth, painless removal of epulis, cauterizing with red hot iron, implantation, etc.

For pulpitis, or peridental inflammation he finds cocaine alone unsatisfactory, but in combination with antipyrine very effectual. He uses the cocaine to anesthetize the parts, the injection of antipyrine being very painful. He uses fifteen grains antipyrine in fifty-five minims of water. The only objection to the use of the latter is a slight induration and swelling, but this only lasts a few days. To insure a fresh solution of cocaine, he prepares a number of packages of one grain each—dissolving one package in twenty drops of distilled water, he uses one-half this quantity for an injection. He attributes his success to the strict antiseptic precautions observed. It bleaches the gums very white and hard, but this is only temporary. Hypodermic injections do not destroy tactile impressions; injected in a vein it produces anæsthesia equal to choloroform. Should the needle strike the bone it must be withdrawn without injecting or necrosis will result. It should never be used if the patient is very much frightened, in which case the patient is liable to become unconscious through vaso-motor disturbance or anemia of the brain. In advanced stages of disease he refuses to administer cocaine, also in patients of great obesity. The antidotes are the inhalation of five drops of nitrite of amyl, and the administration of forty drops of ether in brandy. If a large quantity of cocaine has been used, thirty drops of sulphuric ether may be injected hypodermically at two or three injections.

*Dr. L. G. Noell* offered a supplementary report, enumerating the history and uses of many of the most valuable drugs of the Dental Materia Medica.

*Dr. A. W. Harlan* read a short paper on

THE NEW REMEDIES.

Among these are included creolin with other coal-tar derivatives, antipyrine, antifebrin, new microbes, and the discovery of new remedies for the destruction of their spores going hand in hand. Among the latter he named guiacol, a derivative of wood-creosote, an oily disinfectant of great value where lasting effects or slow disinfection is desired. The aqueous disinfectants permeate the contents of the tubuli more easily than the oily disinfectants, but the integrity of the latter remains unimpaired a much longer time. Guiacol has been sealed up in a pulp chamber forty, sixty, and even seventy days, at the end of that time the contents being found perfectly disinfected. The value of oils has been hitherto unsuspected in this direction.

*Dr. Genese* spoke of the value of the Extract of White Poppies for sedative action, having all the sedative properties of opium, without its soporific effects.

*Dr. Story* spoke of the anæsthetic effects of rapid breathing.

*Dr. J. S. Marshall* asked Dr. Harlan how he demonstated that a cavity was completely disinfected after being sealed up with guiacol.

*Dr. Harlin.*—By filling the root at once, and having no after trouble.

*Dr. Patterson,* Kansas City, thought that hardly satisfactory. What we wanted was to get at the shortest length of time in which germicides would thoroughly disinfect and destroy germs. We should have a scientific and correct demonstration.

*Dr. Sudduth* said the scientific method would be by culture tubes, but Dr. Harlan's method was the most practical.

*Dr. Patterson* thought it mere guess. When new remedies were vouched for, positive evidence of their value should be given.

*Dr. Peabody,* Louisville, Ky., gave his experience in the cure of alveolar abscess by filling root canals with lead. The first case was purely accidental. Ten years ago he had an alveolar abscess on the left superior lateral, which he had treated for eight months, going through the whole range of known remedies, but could not cure it. He decided to extract the tooth, cut off the apex, fill and replace, but the patient desired him to wait until he returned from a two weeks absence. To prevent irritation and keep out food during this time, and also to strengthen the tooth for extraction he concluded a piece of soft lead would answer every purpose. He cut off a strip of lead, rolled it down, cutting it to fit with his knife, and crowded it down into the root canal. At the end of three week's the patient returned, the abscess entirely cured, and there has never been any return.

Having a similar case on a right superior bicuspid he concluded to try lead filling as an experiment. The foramen being open the size of a pin-head, he shaped the lead accordingly, but on inserting it, was conscious that it had passed beyond the foramen, but he did not remove it. He then filled the tooth, using a porcelain face and gold backing to restore the bicuspid. The abscess was entirely cured in five days, and the tooth remained in good condition for two years. Then the crown loosened, and he found that the root was broken in the line of its axis, and he extracted it. He had formed the theory that the apex of the root being bathed in serum which dissolved the lead, forming a salt of lead which had a therapeutic effect curing the abscess. But on extracting the root, he found the lead projecting but with the angles as sharp as the day he cut it with his knife. This knocked his theory out, and he had not formed another. He has practiced this method invariably, for ten years, with almost unvarying success, as is the case with others to whom he has recommended it. The filling is not any more perfect than can be made with other material, but it cures the abscess. He would be glad to learn the therapeusis.

*Dr. J. J. R. Patrick*—Had found that he could confine himself to three therapeutic agents: 1st Corrosive sublimate, 5 grs. to the oz.; 2nd Peroxide of hydrogn; 3rd Nitrate of silver.

The first in the strength given, which is perfectly safe, confined to the root of a tooth, 1-1000 is too trifling; the second for burrowing pus in cancellated bone, for its chemical properties in recombination. It has great penetrating powers, and large affinity for pus. In alveolar abscess there is always more or less pus in the cancellated bone process around the tooth. Peroxide of hydrogen follows it up and clears it all out. After it has been cleaned and dried there is nothing gained in postponing the filling; close it up and keep every thing on the outside.

*Dr. Sudduth*—Said that for a non-poisonous mouth-wash to be used frequently, bichloride of mercury should not be used stronger than one-quarter grain to the ounce. In the strength given by Dr. Patrick it is a dangerous poison, unless strictly confined to a pulp canal.

As to the comparative value of corrosive sublimate and carbolic acid; when we wish to destroy microbes simply, use corrosive sublimate, but in case of chronic abscess, chronic inflammation, just there we want stimulation, and use carbolic acid. In the cases mentioned by Dr. Peabody, he thinks the results due simply to changed conditions, the crisis had been reached.

*Dr. Harroun*, Toledo, said that closing the cavity prevents the entrance of irritants which create the poison that keeps up the trouble. By continually stuffing in one thing after another, we get no cure, like the little boy who kept pulling up the willow switch he planted and so got no tree.

*Dr. L. C. Ingersoll*—Said that men were too apt to ride a bobby to death; go to the widest extremes. No real cure can be had unless we take into consideration all the conditions. It will not always answer to close up at once. There are abnormal tissues around an abscessed root, abnormal vascular activity; this hypertrophied tissue must be absorbed; we must use escharotics and stimulants to destroy that tissue and carry it away. We must also consider the condition of the cementum, as well as of the process and the surrounding membrane. Peroxide of hydrogen is not a stimulant and cannot be trusted alone.

*Dr. W. H. Morgan*—Prefers carbolic acid to all other stimulants; it promotes healthy granulation. When we wish to reduce induration of glandular tissue, promote resolution and absorption, then we want tincture of iodine. Closing the apex prevents the entrance of the thing which does the mischief, which has no further chance; the trouble is from germs passing through the foramen into the soft tissues. Remove the cause and trust to nature. Lead has no therapeutic effect, no chemical action. It merely closes the foramen mechanically.

*Dr. Staples*, Sherman, Texas, said that for years he had tried every thing suggested, treating abscesses three, four, and five months, my idea being the necessity of destroying the abscess sac. Thirteen years ago Dr. Story suggested to me to force in dry oxide of zinc (after treatment with carbolic acid) to close the apex and fill with gutta-percha. I have been doing this for thirteen years, and never fail.

*Dr. A. O. Rawls*—Spoke at length on Pyorrhœa Alveolaris.

He holds to an opinion as to its etiology in opposition to at least one half of the profession viz.: that typical cases of such, are resultant from the action of mercury in some of its remedial forms or from other remedies, or articles of food containing one or more elements common to mercurial medicines. Dr. Rawls holds that the numerous cases of pyorrhœa alveolaris which have been permanently cured are not by any means typical cases of the disease, but only such inflammatory conditions of the gums and immediate peridental membrane as are frequently caused by the deposition of lime-salts or other foreign irritants upon and about these tissues wherein the real susceptibility or predisposition to a typical case does not exist. In typical cases we may use all known antiseptic and germicide remedial agents to no purpose toward a permanent cure but only have in their use such alleviation as would naturally follow the removal of irritants from contact with any part of the system. There are times when different tissues, different organisms are more susceptible to the action of disease producing agents than ever before, and we may have a tendency to disease of certain parts of the system latent for twenty or twenty-five years with no expression of the same and yet when certain conditions are present the least irritant brings about the expression. In true typical pyorrhœa alveolaris when the expression comes the break in the continuity of the nutrient supply is beyond repair by the removal of external irritants germicides or antiseptics.

*Dr. J. C. Story*, Dallas, Texas, said he was glad this subject had been brought up, as he lived in the very hot-bed of the disease, seeing it every day. If mercury is the cause of it, what is the mercury taken for?

*Dr. Rawls*—In the early settlements, the country was all malarial, and mercury, blood-letting and antimony were the principal remedies used. Many salt eaters get it from the element chlorine. They took mercury for the eradication of disease. Subject passed.

PHYSIOLOGY AND ETIOLOGY.

H. A. Smith, American Association; E. S. Chisholm, Southern Association, Chairmen.

*Dr. Smith*—In his report from this Committee, embodied in tabulated form replies received from seventeen of the most prominent operators in

IMPLANTATION

Showing their percentage of success or failure, the causes of failure, the manner of attachment, etc., as follows:

Dr. Cunningham, England, one of the most successful operators, has no faith in Dr. Younger's idea of the revival of the pericementum, and uses only freshly extracted teeth, from healthy young people and kept moist. The question of the mode of attachment is not yet settled. Heitzman and Bodecker's microscopical examination of a tooth that was lost after having been implanted six months, shows no trace left of the pericementum on one side, and but slight remains on the other, with bag-like excavations, zone-like absorption filled with granulation tissue from the alveolus; they deny any living union between the granulations and the dentine. There are three modes of attachment possible—vital union, bony anchylosis, or mechanical attachment. There can be no absolute demonstration except from microscopical examination ef sections of a tooth in the jaw.

Dr. Younger—Attributes failure entirely to want of care on the part of the patient, or the tooth not being held rigidly in position. Dr. W. N. Morrison thinks the only essentials holding the tooth firmly in position by splints and perfect cleanliness. Whether success is sufficient to justify the slight risk of infection remains to be seen. One patient says he would rather have a new tooth implanted every year than wear a plate. Time alone will settle the question of permanency.

*Dr. J. D. Patterson*, Kansas City, read a paper on

PYORRHEA ALVEOLARIS.

He said that the views he had maintained in a former paper as to the catarrhal nature of the disease have been fully confirmed by further observation. He believes it to be a true catarrh having its origin in the oral cavity, and not in the adjoining parts, and extending to adjacent tracts. The mouth used in open breathing is a frequent cause; also irritation from morbid oral secretions. The pathological characteristics exhibited vary with the cause, whether local or systemic, simple or complex. A comparison of the symptoms and special characteristics of catarrh as enumerated by numerous authors, and those of pyorrhea alveolaris, confirming in a very striking manner the views of Dr. Patterson, as shown in the paper. Hypertrophy of tissues, calcific deposits, hyperplasmic membranes, caries of bone, retarded blood circulation, chronic inflammation, loss of function, and other pathological symptoms are exactly the same as manifested in nasal catarrh and in pyorrhea alveolaris.

*Dr. L. Ottofy*—Next read a paper on

THE INCIPIENCY OF DENTAL CARIES.

This paper embodied, in tabulated form, the results of observations on the teeth of children in public schools, the teeth from which the average results given numbering 9,544. The conclusions reached were embodied in a series of large charts—impossible to reproduce in a mere synopsis. In the discussion of the subject of pyorrhea alveolaris,

*Dr. Genese*—Considered boracic acid and aromatic sulphuric acid the most valuable remedies; he prescribes a powder composed of 1 dr. - - - - Boracic acid.
1 oz. - - - - - Precip. chalk.

To be packed in the pockets around the teeth, after they have been thoroughly syringed with aromatic sulphuric acid.

*Dr. Jno. C. Storey*—Uses in the same way a powder as follows:

| | |
|---|---|
| Prep. chalk - - - | 2 oz. |
| Lac sulphur - - - - | 1 oz. |
| Borate soda - - - - | ½ oz. |

He says the sulphur "kills the bugs."

*Dr. J. S. Marshall*—In a very bad case—left upper central incisor, elongated one-quarter of an inch, with the anterior alveolar wall all gone, and also a good share of the posterior, extracted and replanted with success. He found on the root three little points of calcific deposit, in all not as large as a pin's head, and the pericementum partially dead; semi-live pulp. He took an impression in plaster of Paris and made a splint covering the six anterior teeth on the palatine surface and labial edge, which was worn for four months; the tooth not being very firm, it was replaced for four months more ; it has now been off for three months, and he considers the operation a success, with apparent reproduction of alveolus.

*Dr. W. H. Atkinson*—Gave the treatment of a left superior lateral incisor in similar condition, only more so, with an elongation of one-half the length of crown below the line of central and bicuspid, the adjacent teeth all very loose, the gums tumid and discharging pus; there seemed to be no bony socket—no process—only cartilage. Tying the teeth in position, the elongated tooth was trimmed with sharp corundum wheel, to secure proper occlusion ; all debris was washed out and the parts dried with bibulous paper ; aromatic sulphuric acid, full strength, was then dropped in till all pools were filled and soaked, the soft tissues being protected by a strip of bibulous paper under the tongue and inside the lips. Common bicarbonate of soda was used to neutralize the acid on the teeth. A paste of calomel and glycerine was laid in the pockets. If any portions are not "cooked" with sulphuric acid but look like "proud flesh" they must be touched again till they turn grey. If sulphuric acid does not act promptly—if its adaptability is not found satisfactory, then caustic potash and carbolic acid must be used prepared, as follows, to make a perfectly dry paste. 1½ or 2 parts of carbolic acid to 1 of caustic potash crystals are mixed in a mortar which is set in boiling water. This makes a dry paste which can be broken into small pieces with the tweezers and laid in the pockets where it will melt, and do its work effectually with one application. In less than three months there was no appearance of ever having had any trouble, every thing became so firm and healthy. The patient in this case was the wife of the brother of Prof. Dorrance of Ann Arbor, Michigan.

*Dr. W. N. Morrison*—Uses for the removal of deposits from very loose teeth a very delicate long-bladed spatula-forceps ; one beak rests on the incising end of the tooth to steady it, the closing motion of the forceps scaling the deposits off the root.

*Dr. J. Taft*—Said that the great difficulty in these discussions is that time is too short for full consideration of the subject. We are told by one what was done, by another what treatment was given, but what is indicated by that? To understand a case perfectly means more than to look at it and see that the gums are separated from the necks of the teeth, more than that deposits are present, more than that there are diseased tissues around the teeth, and in the walls of the sockets; it means more than all this. What is the organization of the patient? What is his general condition? Is nutrition good? Can he resist irritants? What are the accidental conditions of health, or is there any digression from health, and how far? All this, and more, is embraced in the diagnosis of a case. What is required? Are there deposits of foreign substances between the roots and the gum constituting a source of irritation? Is there undue pressure on the tooth? Is it partially loosened? Is it overtaxed in mastication, or is it suffering from not being used? All these conditions, if existing must be remedied.

The gum takes on diseased conditions from various causes. It is still an unsettled question how much these are influenced by the presence of organisms. The varying susceptibility of tissues is also a factor, some being much more susceptible than others, yielding readily to the least irritant. To secure the best remedial results all these points must be considered.

All irritants must be removed. If there is a diseased condition of the alveolar margin, organisms must be destroyed, and diseased tissues dissolved away, either by aromatic, sulphuric acid, full strength, or preferably pure sulphuric acid 1 to 7 parts of water. This cauterizes the dying, half dead, or dead tissues lining the socket. There can be no restoration to health while deteriorated tissue remains. Sometimes chloride of zinc or caustic potash and carbolic acid acts better than sulphuric acid. A good clean surface of healthy living tissue must be secured, from which plasm can flow out and granulation take place. Then adhesion to the root will take place, in some cases tardily, in others with remarkable rapidity. We must be satisfied when we have put the case on the road to reparation, and then let it alone—do not overtreat.

One man said he had lost all patience with sulphuric acid because he had applied it ten times, and the case was worse than when he began.

Was it any wonder? It should rarely be applied more than once. Get the soft tissues so that healthy plasm will flow and organize and it will return to health.

*Dr. J. C. Story* said he had done all that, in just that way, and still they don't get well; they seem to improve, but six months, or in twelve months, they come back as bad as ever.

*Dr. Geo. J. Friedrichs* asked if the mouth can be made aseptic, and how long it will remain aseptic?

*Dr. Taft.*—As long as cleanliness is maintained, and healthy plasm is thrown out. In Dr. Story's returning cases, perhaps the deposits were not thoroughly removed to the apex, or the mouth was allowed to become unclean, or there was a low condition of the nutrient forces. A physician never guarantees a man to be free from disease in the future; he is always liable to become sick again.

On motion, the subject was passed.

ANATOMY, PATHOLOGY, AND SURGERY.

Dr. Morgan Adams, Southern Association; Dr. T. W. Brophy, American Association, Chairmen.

Dr. Brophy made the report for this committee. He said that marked advances had recently been made in surgery, especially in the transplantation of tissue, from man to man, and from the lower animals to man, especially from rabbits to man.

Among the most remarkable successes were the transplantation of nerve-tissue, with restoration of nerve function, and the transplantation of cornea with restoration of sight. For success in these and other operations, such as bone or skin grafting, tooth implantation, etc., there must be complete absence of catarrhal or inflammatory conditions and the maintenance of strict aseptic conditions.

*Dr. J. S. Marshall.*—Gave the history of a case of successful grafting of rabbit's bone in his own practice, as reported to International Medical Congress (see REGISTER, December, 1887, page 615). This, he said, was the first case of the kind on record. He also exhibited a tooth—left superior central incisor—in the labial aspect of which, midway between the gum and the cutting edge, is what appears to be a sequestrum, something hitherto never seen in dental tissue. He requested Dr. Sudduth to make a microscopical examination and report upon it.

Dr. Marshall also reported a fatal case of alveolar abscess. The subject had been operated on at the Mercer Hospital, to get rid of the effusion, the cut having been made in the side of the neck, about an inch below the jaw; in the operation the facial artery had been cut, with severe hemorrhage. When Dr. Marshall first saw the case, April 26th, an offensive discharge was oozing from the cuts. The face was very much swollen and infiltrated full. He was called to extract an inferior wisdom tooth which was much decayed, and was very sore even to the touch of the tongue. The report gives the daily treatment of the case, with temperature, pulse, respiration, etc. After the extraction of the wisdom teeth, May 2d, there was marked improvement, the patient sitting up in a chair. On the 6th, large masses (4 oz.) of necrotic tissue were removed from the neck. On the 8th of May the ligature of the artery sloughed off, and an extensive hemorrhage weakened the patient seriously. On the 11th another hemorrhage occurred and the patient died that night from exhaustion. Dr. Marshall attributes his death to "alveolar abscess allowed to run too long."

*Dr. Atkinson* thought the extraction of the wisdom teeth, in the necrotic condition of the soft tissues, the stepping stone to death.

*Dr. J. Y. Crawford* was satisfied that sufficient regard was not paid to the influence of the wisdom teeth in leading to serious consequences. He was surprised to hear Dr. Atkinson criticize the removal of the wisdom teeth under the condition described; he could not see that any other procedure than the removal of the offending member was possible. The decayed wisdom tooth was the exciting cause of the alveolar abscess.

*Dr. J. D. Patterson* thought the chief factor in death was the blundering operation of the surgeon who cut the artery, exhaustion resulting from the hemorrhages, and not from the discharge from the abscess.

*Dr. W. X. Sudduth* spoke at some length on the subject of implantation. He said that the older operations of replantation and transplantation had been tried and condemned; they had failed because the attempt was made to replace a tooth in the midst of diseased territory, where there was, or had been abscess or necrosis; it was a retrograde process, with everything against it. In transplantation an old tooth not worth crowning, perhaps an old root that had long borne a pivot-crown, was removed and another placed in its diseased socket. With implantation everything is different; a new cavity is made in healthy tissues; there is no retrograde tendency to overcome; from a wound made in healthy tissues we have healthy exudations, forming fibrous tissue; bone cells are thrown out, forming new bone. If inflammation is kept at a minimum, only sufficient for stimulation, the tooth is fastened in its place—not through vital connection but by bony anclylosis. To Dr. Younger is due the credit of having originated the idea, but Dr. Kirk has placed it on a higher scientific basis. Our present knowledge of antisepsis is one great factor in the possibilities of success. The attachment of the implanted tooth is very firm, but its method is still an open question for the reason that successful cases cannot be investigated; no one is willing to part with one; it is to be hoped that experiments will be made with the lower animals that this question may be settled. The peculiar resonance of an implanted tooth would indicate anchylosis. There is no soft cushion of pericementum for this is not revived, but the tooth is fixed solid in the jaw. For this reason it receives more shock from mastication, and if the vital forces are lowered that tooth could probably be less able to resist, though this is only theory. If the tooth remains firm for three, four, six years, why is not this a true and correct surgical operation? It is simply transplanted tissue.

*Dr. W. H. Morgan* said he was not prepared to accept the operation. He feared serious results from the introduction of dead matter into the animal organism. When the tooth is retained for a time it is encysted—held by tissues drawn into the inequalities of the cementum—but it is scar tissue which will not be permanently tolerated. Nature will eventually pull down this foreign matter and eject it like a splinter. We are justified in expecting that every case will end in that way sooner or later.

We have not given sufficient attention to erupting wisdom teeth. The large overlap of gum on the posterior surface is often more or less diseased ; an ichorous discharge from this, dripping into the throat, gives trouble.

*Dr. J. S. Marshall* said that he had never known Father Atkinson to listen so poorly ; he charged that the extraction of the diseased tooth had as much to do with the death of the patient as anything else, whereas the report had distinctly stated that the patient really recruited after the extraction until the sloughing of the ligature caused severe hemorrhages.

*Dr. Atkinson* replied that he loved the young man too well to let him go on in error without an attempt to set him right. The peculiar decrepitation described did not indicate the presence of liquids, which don't act that way, but of gasses, the tissues were poor, not properly organized ; something kept up a ferment, setting free oxygen and hydrogen or sulphur and hydrogen; elements were set free that make crepitation; that is not the movement of fluids. According to the report four ounces of bad smelling matter were taken away; how came that sloughing on both sides, with but one wisdom tooth diseased ?

*Dr. Marshall* replied that it had extended by burrowing ; the swelling had extended to both sides before he saw him the first time, and had been opened on both sides. He was well aware that it was necessary to be cautious about extracting where there is necrosis of the soft tissues, or high pericementitis. He thought Dr. Atkinson had jumped to conclusions without a basis.

*Dr. Patrick* asked where the thermometer was placed in taking the recorded temperature ?

*Dr. Marshall.*—In the axilla and the mouth.

*Dr. Brophy* said it had not been proved that the wisdom tooth was the original cause of the disease. He thought the extraction of the wisdom teeth, one not being abscessed, was scarcely justifiable in the broken down condition of the surrounding tissues, giving chance for the absorption of septic matter, of poisoning from septicemia. He would not say it had led directly to the death of the patient, but under the circumstances he would not have extracted. He thought the direct cause of death was the great debility from loss of blood from the arterial hemorrhage. He is not an ardent advocate of implantation but hopes it may prove a success. An implanted tooth does become a fixture, and must let down finally.

*Dr. Atkinson* cautioned against the too free use of peroxide of hydrogen, which moves in the line of least resistance and sometimes leaves the mouth bloodless, entirely white from its strong affinities for pus, sanies, ichor. If the parts are thoroughly cleansed with carbonate of soda (washing soda), it can be used with safety.

*Dr. Beach* thinks implanted teeth are held in place by an encasement of bone, but thinks the union of dead bone and living bone an impossibility.

*Dr. J. S. Marshall* said it was an easy matter to report our successes, but it require some courage to report a case of failue and death.

*Dr. Abbott.*—If we had no failures, both we and our patients would be better off. In implantation there is no union of tissues, either by gomphosis nor anchylosis, otherwise than as a nail is held in a board. A socket is dug out and shaped to fit the root so closely that force is often necessary to remove it, there is usually no necessity for ligatures; the inflammation produced in the bone, in digging out the socket, produces granulations which force their way into the surface of the root of the tooth, the substance of which is dissolved out by that process; if it were not perfectly tight there would be millions of organisms and decomposition would rapidly follow. To remove it thousands of fibres of granular tissue which have grown into the dead bone must be torn apart.

*Dr. Morgan.*—Inflammation does not necessarily produce any dissolving out; there is a condition of irritation which is benign, but is not inflammatory.

*Dr. Abbott.*—It is difficult to determine where irritation stops and inflammation begins.

*Dr. H. A. Smith.*—If the tooth is driven in forcibly, a high grade of inflammation will result, causing disintegration. An accurate fit is desirable but not to necessitate driving in forcibly.

*Dr. Sudduth.*—If the union is by fibrous tissue, why does it give out that sharp resonant sound?

*Dr. Peabody* asked how a tooth with no constriction at the neck would be held in?

*Dr. Abbott* did not think it would be retained.

*Dr. Catching* said he had had positive proof of anchylosis, in a tooth which had been implanted by Dr. E. S. Chisholm; to remove it, it had been necessary to nip off the process with alveolar forceps.

*Dr. Taft* said that it was very true that there would be no union between living and dead tissues, yet he did not consider the implanted tooth dead in the truest sense; it is structurally prepared for the reception of organic material the cementum at the surface is structurally ready; organization takes place in the interstices, as in sponge graft; the sponge is not any more living matter than is the cementum of the tooth; it is simply fitted to receive the plasm thrown out, which displaces the material already there, replacing it by living tissue; nutrition being afforded, firm attachment takes place; calcific material is deposited and firm bony union takes place. No other hypothesis can explain the result, but it is a theory which needs demonstration and proof.

Subject passed.

Dr. Taft moved that all papers yet on hand be read, notes taken and discussed afterwards. Too often the last papers are read hurriedly, or by title, or not at all.

PROSTHETIC DENTISTRY, METALLURGY, CHEMISTRY.

*Dr. J. R. Knapp*—Reported from this committee that from the vast extent of the field they were called on to survey, but a partial list of what had been suggested or effected could be given. Numerous publications of great value to the profession have appeared during the past year. The third volume of the American System of Dentistry completes a work which is an honor to the country and to the profession. Among other valuable publications enumerated are Professor Clifford Mitchell's Special Chemistry, Dr. Haskell's Handbook for the Laboratory, Buxton's Manual on Anæsthetics, Essig's Metallurgy, the 4th edition of Richardson's Mechanical Dentistry, Evans' Crown and Bridgework, Andrieu's Progress Dentaire. The report also included a history of the dental use of aluminum, and of cocaine, the sense of the committee being unfavorable to the use of the latter, supported by numerous instances of dangerous results.

*Dr. Genese*—Read a paper on

RUBBER

Which, he said, had undoubtedly come to stay; the dental student should therefore be taught the proper way to use this material so as to obtain the best results, and avoid the objectionable features. He said he was not an advocate for cheap material, or cheap work. Rubber and its prototype amalgam, must be used more or less, and both should receive the best treatment at our hands.

*Dr. J. S. Marshall*—Offered a resolution to the effect that the tariff on imported dental and surgical goods works a hardship upon the profession and the public, therefore,

*Resolved*, That we memorialize Congress to abolish all duties upon imported dental and surgical instruments, apparatus and supplies. The Secretaries of the Associations were by the resolutions instructed to forward them to the proper authorities in Congress, and each member of the Associations requested to use his influence with the congressman from his district in this regard. The vote in the affirmative was almost unanimous.

DENTAL EDUCATION AND LITERATURE.

*Prof. J. Taft*—Made a brief verbal report from this committee, announcing five papers, one each from Drs. L. Ottofy, W. H. Atkinson, L. C. Ingersoll, B. Holly Smith, and D. R. Stubblefield.

*Dr. Ottofy's*—Paper was largely statistical, reviewing the number of colleges, number of graduates, discussing also the mooted question of length of time, number of terms, etc., requisite for preparation of the average student for graduation.

*Dr. Atkinson's*—Paper was on the proper basis for teaching

TERMINOLOGY.

*Dr. B. H. Smith's*—Dealt with the best methods of training the dental student to enable him to attain the high standard set before him as a recognized specialist in medicine—advocating both the extension of the term of study, and increased severity in prerequisites for admission to the Dental College.

*Dr. L. C. Ingersoll's*—Paper discussed

METHODS IN DENTAL COLLEGE EDUCATION,

Advocating the method of questions and answers, or the recitation system, in preference to the lecture system, which was adopted when text books were few and rare and accessible only to the professors.

*Dr. Stubblefield's*—Paper was a general review of the whole subject of

DENTAL EDUCATION OF YESTERDAY, TO-DAY, AND TO-MORROW.

These papers were followed by one from Dr. Geo. J. Friedrichs on DENTAL HYGIENE.

In the discussion which followed Dr. J. C. Storey gave a curious instance of the toxicological effects of cocaine. In using a four per cent. solution of cocaine for spraying the throat, some was accidentally spilled on the floor; the next morning three dead rats were found on the spot. As a further test, some more was purposely spilled, and the next morning four more dead rats were found. From that he concluded that it was very liable to "kill folks" too. There had been some ugly cases of poisoning, from its use in Dallas and he would not use it in his practice.

*Dr. Friedrichs*—Said he had been using cocaine in hypodermic injections for two years, and his son was using it freely in hospital clinics, extracting from fifteen or twenty to forty and fifty teeth daily, without seeing any of the peculiar effects described by some authors. (At this point a telegraphic communication was read inviting the members of the Associations to attend the meeting of the Mississippi Valley Medical Society, Sept. 25. On motion of Dr. Catching the Secretary was instructed to return the thanks of the Association for this courtesy).

*Dr. Sudduth*—Said he had experimented freely with cocaine in strength varying from two to fifty per cent. without seeing any of the bad effects reported elsewhere, though in certain cases its action was not satisfactory. As a rule he considered it as safe as any anæsthetic that can be used.

*Dr. Harlan*--Suggested that the authors of the paper read restate the main points as an aid to intelligent discussion.

*Dr. Ingersoll*—Said he had said his say and would prefer to hear some one say something in opposition. Although his views were well known, he had never before formulated them in a paper for a society. Dentistry is not, in the common acceptation of the term, a specialty of medicine. Dentists, as a rule, do not read medical literature. The faculties of medical colleges are not prepared to teach dentistry. Dentistry is an independent science. The dental student who studies dental anatomy is not studying medicine any more than the school-girl who studies "anatomy and physiology."

He studies chemistry as a natural science, not as medicine. You can not put a dental student in the same class with a medical student and do him justice. He has not time to go over so much ground, and he only wants that which is applicable to his work as a practical dentist. Another point; our clinical rooms should not be called infirmaries; patients do not come there to be treated for infirmities; let us drop this unmeaning term and say clinical or operating room. As to the special points of my paper you can all read it when it is published.

*Dr. W. H. Morgan*--Said that the most radical view taken in the paper was as to the manner of imparting knowledge. He would fully subscribe to what had been said as to dentistry being an independent profession. If every medical college were wiped out to-day, and every strictly medical book burned, dentistry would still be left intact; it would not make a ripple in the current of its progress.

The method of teaching by recitation was largely adopted in the institution with which he is connected; it is the most thorough method known, though the didactic method is the best adapted to certain topics. Prosthetic dentistry should be confined entirely to recitation and demonstration. It is the difference between reading of a country and traveling through it. Forty descriptions are not so good as one sight.

*Dr. Guilford*--Thought the recitation method too primary for college students who should not be treated like little children.

Facts should be laid before them, and though they may not grasp all, *the quiz* will show how much they have understood. This, with the clinic room and the practice is the true method. Our colleges should not receive students who are not prepared to profit by this method. We have no text books suitable for use in the recitation method. Advances are so rapid that text books could not be multiplied to keep up with it. The teachers are posted through voluminous books of reference, and are able to give broader views by didactic teaching.

*Dr. Taft*—Said that all this discussion about the relation of medicine and dentistry was unprofitable; it will not be changed by it, but it will be adjusted by other means. Dentistry is an important branch of the healing and restorative art. It can not be independent of medicine or any branch of it. The more we know about general constitutional conditions and their effect upon the oral cavity the better we are prepared for our special and legitimate work. Medicine and dentistry are so inter-blended and inter-woven that it is utterly impossible to separate them; it is therefore a most unprofitable matter of discussion. As to the best method of teaching, it ought to be something more than mere talk. It would be greatly to our advantage if more uniform methods could be adopted. The National Association of Dental Faculties has not yet settled upon any definite method of procedure in teaching, but it has muc hunder consideration—matters looking to improvement and uniformity. It would be well if we could have a normal institute of dental teachers, and deliberately discuss modes and methods; through interchange of visits much might be learned. It is important that methods be made uniform as far as possible.

As to the details of the methods of teaching these are more for the college faculties than for bodies like this—but after, all these things are or ought to be of interest to every progressive member of the profession.

How far it is possible to bring all colleges on to the same uniform method of procedure time only can decide. But that such uniformity is desirable, and for the best interests of all no one will deny.

*Dr. Atkinson*—Said that the schools, both medical and dental, fail to go into first principles, except in name. The truth is revealed by surroundings independent of the teachers; the best teacher is he who so points it out that the student may discover it for himself. The best way to lead the pupil is to lead him very simply. The highest scholar may be classed with the beginner as he recognizes with humility how little he has conquered of that which seems almost within his grasp. The papers we have heard will prove more profitable when read at leisure than when discussed so imperfectly. Those who can look back and remember the talk we had in our first meetings can see how grand has been our progress, and that we are now worthy to be recognized by the world as *healers.*

*Dr. Friedrichs*—Being asked for the principal points in his paper said that they were, *first*, the query as to the best substitute for mother's milk; *second*, the all-importance of cleanliness and the means of antisepsis; *third*, the consequences of neglect of alveolar abscess.

*Dr. Atkinson*—Said he was glad of the opportunity of making an honest confession. He had not given Dr. Friedrichs credit for the ability to write such a paper; either he had done him a great wrong or the angels wrote it for him—which he kicks at. What is health? What is disease? We have organic consciousness of *dis—ease*; we have tissual consciousness; corpuscular consciousness; molecular consciousness; all these must be awakened ere the hungry soul can be made fat. If all the organs are not in proper order, serious consequences will result to the organism. Tonics enter into the tissues and make them strong; stimulants wake up the energies, but do not enter into the tissues.

*Dr. Patrick*—Said there were positive facts in every science that medicine is built upon, but medicine itself is not a science; it is only theory and practice. If teachers would confine themselves to facts, there are demonstrated facts enough in every department that the colleges teach, to occupy four times the time that is given to dental college education.

The hour for adjournment having arrived the discussion was declared closed.

After feeling words of retrospection and farewell by Drs. Abbott and Catching, on motion of Dr. Taft, a resolution of thanks to the officers who had so efficiently conducted affairs as to make the meeting a credit to the two associations and to the profession, and one long to be remembered for its pleasures and its success, was unanimously carried.

The Joint Meeting of the American and Southern Associations then finally adjourned at 10 p. m., Aug. 31, 1888.

